# Junctional adhesion molecule-A is dispensable for myeloid cell recruitment and diversification in the tumor microenvironment

**DOI:** 10.3389/fimmu.2022.1003975

**Published:** 2022-12-01

**Authors:** Máté Kiss, Els Lebegge, Aleksandar Murgaski, Helena Van Damme, Daliya Kancheva, Jan Brughmans, Isabelle Scheyltjens, Ali Talebi, Robin Maximilian Awad, Yvon Elkrim, Pauline M. R. Bardet, Sana M. Arnouk, Cleo Goyvaerts, Johan Swinnen, Frank Aboubakar Nana, Jo A. Van Ginderachter, Damya Laoui

**Affiliations:** ^1^ Myeloid Cell Immunology Lab, VIB Center for Inflammation Research, Brussels, Belgium; ^2^ Laboratory of Cellular and Molecular Immunology, Vrije Universiteit Brussel, Brussels, Belgium; ^3^ Laboratory of Dendritic Cell Biology and Cancer Immunotherapy, VIB Center for Inflammation Research, Brussels, Belgium; ^4^ Laboratory of Lipid Metabolism and Cancer, KU Leuven, Leuven, Belgium; ^5^ Laboratory for Molecular and Cellular Therapy, Vrije Universiteit Brussel, Brussels, Belgium; ^6^ Division of Pneumology, CHU UCL Namur (Godinne Site), UCLouvain, Yvoir, Belgium; ^7^ Division of Pneumology, Cliniques Universitaires St-Luc, UCLouvain, Brussels, Belgium

**Keywords:** junctional adhesion molecule-A, JAM-A, JAM-1, F11R, monocyte, extravasation, tumor-associated macrophage, interleukin-1

## Abstract

Junctional adhesion molecule-A (JAM-A), expressed on the surface of myeloid cells, is required for extravasation at sites of inflammation and may also modulate myeloid cell activation. Infiltration of myeloid cells is a common feature of tumors that drives disease progression, but the function of JAM-A in this phenomenon and its impact on tumor-infiltrating myeloid cells is little understood. Here we show that systemic cancer-associated inflammation in mice enhanced JAM-A expression selectively on circulating monocytes in an IL1β-dependent manner. Using myeloid-specific JAM-A-deficient mice, we found that JAM-A was dispensable for recruitment of monocytes and other myeloid cells to tumors, in contrast to its reported role in inflammation. Single-cell RNA sequencing revealed that loss of JAM-A did not influence the transcriptional reprogramming of myeloid cells in the tumor microenvironment. Overall, our results support the notion that cancer-associated inflammation can modulate the phenotype of circulating immune cells, and we demonstrate that tumors can bypass the requirement of JAM-A for myeloid cell recruitment and reprogramming.

## Introduction

Junctional adhesion molecule-A (JAM-A, also known as JAM-1 and F11R) is a single transmembrane domain protein that is expressed on endothelial cells, epithelial cells, immune cells and platelets (1). JAM-A on monocytes and neutrophils is required for diapedesis at sites of inflammation by promoting de-adhesion and polarized cell movement during transmigration through the endothelium (2, 3). In neutrophils, JAM-A exerts this function by co-clustering with β_1_ and β_2_ integrins on the membrane upon chemotactic stimuli and promoting their internalization, thereby facilitating uropod retraction and directional migration (2, 4). JAM-A in neutrophils is also required for the activation of Rap-1 (4), a key regulator of cell adhesion and migration-related processes, such as cell spreading and inside-out integrin activation (5). The mechanisms by which JAM-A controls monocyte diapedesis are less well understood. JAM-A-deficient monocytes adhere stronger to the β2 integrin ligand ICAM-1, indicating that JAM-A modulates integrin signaling in this cell type as well (3). Endothelial JAM-A may also direct the movement of leukocytes through cell-cell junctions by interacting with JAM-A or LFA-1 on myeloid cells (6, 7).

Accordingly, deletion of JAM-A in immune cells reduces monocyte and neutrophil accumulation in various disease settings in mice, including atherosclerosis, peritonitis and ischemia-reperfusion injury of the heart (2, 3). Pro-inflammatory pathways are often coopted in cancer to induce accumulation of monocyte-derived macrophages and neutrophils in tumors, and in turn, these cells contribute to disease progression and therapy resistance (8, 9). This raises the question whether JAM-A signaling could promote the recruitment of monocytes and neutrophils to tumors, and thereby contribute to disease progression. At present, the role of JAM-A was only investigated in tumor-associated dendritic cells, whereby JAM-A deficiency in these cells paradoxically increases their ability to accumulate in tumors for reasons that are not fully understood yet (10).

JAM-A not only mediates cellular interactions, but also connects to downstream signaling pathways partly *via* its intracellular domain and partly through interacting with integrins (1, 11). Hence, JAM-A could potentially modulate cellular functions beyond migration and adhesion in myeloid cells. This is supported by the observation that macrophages isolated from the brain of JAM-A-deficient mice show gene expression changes, enhanced phagocytic capacity and promote the proliferation of glioblastoma cells *in vitro* (12).

Notably, whole-body JAM-A-deficient mice show increased vascular permeability and compromised intestinal epithelial barrier due to impaired tight junction function, which alters systemic immune homeostasis (13–16). Hence, generation of lineage-specific knock-out mouse models is essential to avoid these systemic effects and unequivocally define the role of JAM-A in the regulation of myeloid cell recruitment and phenotype in cancer.

In the current study we set out to address these knowledge gaps by characterizing the expression of JAM-A in circulating and tumor-infiltrating myeloid cells and by interrogating its role in myeloid cell recruitment and phenotypic diversification in tumors using mice with myeloid cell-specific JAM-A-deficiency.

## Materials and methods

### Mice

All experiments were performed with age-matched female mice. C57BL/6J mice were from Janvier, UBI-GFP and LysM-Cre mice were from Jackson, *Il1b* ^-/-^ mice were provided by François Huaux (UCL, Belgium), C57BL/6-MMTV-PyMT mice were provided by Massimiliano Mazzone (KU Leuven, Belgium) and *F11r* ^flox/flox^ mice were provided by Rory Koenen (Maastricht University, The Netherlands). All procedures followed the guidelines of the Belgian Council for Laboratory Animal Science and were approved by the Ethical Committee for Animal Experiments of Vrije Universiteit Brussel (licenses 14-220-26, 19-220-8).

### Cell lines and tumor models

LLC and Py8119 cells were from ATCC. LLC cells were maintained in DMEM (Gibco) supplemented with 10% (v/v) heat-inactivated fetal calf serum (FCS; Capricorn Scientific), 300 μg/ml L-glutamine (Sigma), 100 units/ml penicillin and 100 μg/ml streptomycin (Gibco). For Py8119 cells, the medium was F12K (Gibco) and MITO+ serum extender (1:1000, Corning) was added in addition to the supplements above. eGFP-expressing cell lines (produced as described below) were cultured like the parental cell lines. For LLC/LLC-eGFP tumor implantation, 3×10^6^ cells were injected subcutaneously into the right flank of mice in 200 μl of HBSS. For Py8119/Py8119-eGFP tumors, 10^6^ cells were injected into the left 4th mammary fat pad in 50 μl of HBSS mixed with Growth Factor Reduced Matrigel (Corning) in a 1:1 ratio. Tumor volumes were determined by caliper measurements and calculated using the formula: V = π × (d^2^ × D)/6, where d is the shortest diameter and D is the longest diameter. Tumor, blood, spleen, bone marrow and lung were collected when the tumor reached maximum 1500 mm^3^ volume.

### Lentiviral transduction of cell lines with eGFP

The packaging plasmids GAG and REV, the VSV.G encoding plasmid VSV and the pCCLsinhPGK-eGFP-wpre transfer plasmid were kind gifts from Prof. Brian Brown (Mount Sinai Icahn School of Medicine, NY). In order to prepare third generation lentiviral vectors (LVs), HEK 293T cells were plated at 15x10^6^ cells per 175 cm^2^ culture flask (Falcon). The next day, these cells were transfected using 130.5 μg polyethyleneimine (Polysciences, Eppelheim, Germany) with 12.5; 6.5; 9 and 37.5 μg of the GAG, REV, VSV and eGFP encoding plasmid, respectively per culture flask. LV-containing supernatant was collected on days 2 and 3 after transfection and concentrated by ultracentrifugation @ 22000rpm for 1.5 hr. LV stock characterization and titration was performed by transducing HEK293T cells with dilution series of the LV stock and flow cytometric evaluation of eGFP expression three days after transduction. Lentiviral transduction of cells was performed as described previously (17). Briefly, 10^5^ cells/well were seeded in a 6 well plate. LV particles were resuspended in culture medium together with protamin-sulphate (1:1000) and cells were incubated with this transduction cocktail overnight. The amount LV was chosen to achieve a MOI (Multiplicity of Infection) of 10.

### Clinical specimens

Fresh tumor tissue was obtained from 3 patients with NSCLC undergoing surgical resection at Cliniques universitaires Saint-Luc-UCLouvain (Brussels, Belgium). The study was approved by the local ethics committees of the Vrije Universiteit Brussel and Saint-Luc-UCLouvain and all patients provided informed consent after the nature and possible consequences of the study had been explained. After resection, samples were immediately transported on ice to the research facilities at Vrije Universiteit Brussel.

### Blood collection and tissue dissociation

Blood was collected from mice in 1 mL syringes containing 0.5 mM EDTA. Tumors, lungs and lymph nodes were excised, minced into small pieces, incubated with 10 U/mL collagenase I (Worthington CLSS-1), 400 U/mL collagenase IV (Worthington CLSS-4), and 30 U/mL DNase I (Worthington DCLS) in RPMI for 30 minutes at 37°C, squashed, and filtered. Spleens were mashed through a cell strainer, bone marrow was flushed out from the femurs into RPMI. Human tumors were dissociated using the method applied for mouse tumors. All single-cell suspensions were treated with Ammonium–Chloride–Potassium erythrocyte lysis buffer.

### Flow cytometry and cell sorting

Cells were resuspended in HBSS with 2 mM EDTA and 0.5% (v/v) FCS. To prevent nonspecific antibody binding to Fcγ receptors, cells were preincubated with CD16/CD32-specific antibody (clone 2.4G2). Cell suspensions were then incubated with fluorescently labeled antibodies diluted in HBSS with 2 mM EDTA and 0.5% (v/v) FCS for 20 minutes at 4°C and then washed with the same buffer.

The following anti-mouse fluorochrome-conjugated antibody clones were used in the study: JAM-A (REA854), CD45 (30-F11), CD11b (M1/70), Ly6G (1A8), SiglecF (E50-2440), MHC-II (M5/114.15.2), Ly6C (HK1.4), CD43 (S7), F4/80 (CI:A3-1), CD11c (HL3), NK1.1 (PK136), CD19 (B4), CD4 (GK1.5), CD8 (53-6.7), CD31 (390), β_1_ integrin (HMβ1-1), β_2_ integrin (M18/2).

For flow cytometry analysis of human tumors, the following anti-human fluorochrome-conjugated antibody clones were used: JAM-A (REA605), CD45 (2D1), CD66b (G10F5), CD16 (3G8), Siglec8 (7C9), HLA-DR (L243), CD14 (M5E2), CD163 (GHI/61), CD11c (3.9), CD31 (WM59), CD3 (HIT3a), CD56 (5.1H11), CD4 (OKT4), CD8 (RPA-T8), CD19 (HIB19). 7-AAD or Fixable Viability Dye eFluor 506 (eBioscience) was used to exclude dead cells.

Cell fixation and permeabilization for staining of intracellular proteins was performed using a fixation/permeabilization kit by eBioscience (00-5523-00).

Flow cytometry data were acquired using BD FACSCanto II (BD Biosciences) and analyzed using FlowJo software. Cell sorting was performed using BD FACSAria II (BD Biosciences).

### Single-cell RNA sequencing

Sample preparation for sort: To limit dissociation-induced gene expression, tumors were dissociated in the presence of actinomycin D (ActD), following a protocol described before (18). The concentration of ActD varied as follows: 30 μM during cutting, 15 μM during enzymatic digestion and 3 μM for all remaining steps. After tissue dissociation and antibody staining as described above, 45 000 7-AAD^-^CD45^+^ cells pooled from three size-matched tumors per genotype were sorted into RPMI with 10% (v/v) FCS, 300 μg/ml L-glutamine, 100 units/ml penicillin, 100 μg/ml streptomycin, 1% (v/v) MEM non-essential amino acids (Gibco), 1 mM sodium pyruvate (Gibco), 0.02 mM 2-mercaptoethanol (Sigma) and 3 μM ActD.

Post-sort sample preparation: Sorted cells were centrifuged for 6 min at 4°C at 400 g and resuspended in PBS with 0.04% bovine serum albumin in a final concentration of 1000 cells/µl.

Library preparation: Single-cell suspensions were loaded on a GemCode Single Cell Instrument (10x Genomics) to generate single-cell gel beads-in-emulsion (GEM). GEMs and scRNA-Seq libraries were prepared using the GemCode Single Cell 3’ Gel Bead and Library kit, version 3.1 (10x Genomics, No. 1000121) and the Chromium i7 Multiplex Kit (10x Genomics, No. 1000213) according to the manufacturer’s instructions. Briefly, GEM reverse-transcription incubation was performed in a 96-deep-well reaction module at 53°C for 45 min, 85°C for 5 min and ending at 4°C. Next, GEMs were broken and complementary DNA (cDNA) was cleaned up with DynaBeads MyOne Silane Beads (Thermo Fisher Scientific, No. 37002D) and SPRIselect Reagent Kit (Beckman Coulter, No. B23318). Full-length, barcoded cDNA was PCR amplified with a 96-deep-well reaction module at 98°C for 3 min, fourteen cycles at 98°C for 15 s, 67°C for 20 s and 72°C for 1 min, one cycle at 72°C for 1 min and ending at 4°C.

Gene expression library construction to generate Illumina-ready sequencing libraries was performed after cleanup with the SPRIselect Reagent Kit and enzymatic fragmentation, by adding R1 (read 1 primer), P5, P7, i7 sample index and R2 (read 2 primer sequence) *via* end-repair, A-tailing, adapter ligation, post-ligation SPRIselect cleanup/size selection and sample index PCR. The cDNA content of pre-fragmentation and post-sample index PCR samples was analyzed using the 2100 BioAnalyzer (Agilent). Sequencing libraries were loaded on an Illumina NovaSeq 6000 flow cell with sequencing settings according to the recommendations of 10x Genomics (read 1: 26 cycles; read 2: 98 cycles; index i7: eight cycles; index i5: no cycles).

### Single-cell RNA sequencing data analysis

The Cell Ranger pipeline (10x Genomics) was used to perform sample demultiplexing, mapping to the reference genome (mouse mm10), barcode processing, unique molecular identifier filtering and generation of gene by cell expression matrices. The average reads per cell was 17,476 and 19,394 for the *F11r* ^fl/fl^Cre^-^ and the *F11r* ^fl/fl^Cre^+^ library, respectively. In total, transcriptomes for 25,422 CD45^+^ cells were generated (*F11r* ^fl/fl^Cre^-^: 13021 cells; *F11r* ^fl/fl^Cre^+^: 12401 cells). The gene expression matrices were further preprocessed and filtered using DropletUtils, Scater and Seurat R packages. Barcodes associated ambient-RNA-containing droplets were filtered out using the EmptyDrops utility of DropletUtils. This method identifies cell-containing droplets, by testing each barcode’s expression profile for significant deviation from the ambient profile. Further, outlier cells were identified based on three metrics (library size, number of expressed genes and proportion of expressed mitochondrial genes); cells were tagged as outliers when they were two median absolute deviations distant from the median value of each metric across all cells. Low-abundance genes were removed following the recommended workflow of Lun et al. (19). A threshold based on the distribution of log-mean counts per gene across all genes was chosen for both libraries (0.007 mean counts). The raw counts were normalized by the default normalization method of Seurat that scales the gene expression of each cell by the total expression per cell, multiplies it by a scale factor (10,000), and log-transforms the result. Highly variable genes were detected using FindVariableFeatures functionality of Seurat and the data was scaled and centered per gene. Subsequently, the scaled highly variable gene counts were used for unsupervised dimensionality reduction and clustering. Enriched genes per cluster were identified using the Seurat FindAllMarkers function (min.pct = 0.1, logfc.threshold = 0.25). Differential expression analysis within cell clusters was performed using Wilcoxon Rank Sum test through the Seurat FindMarkers function. *P* value adjustment was performed using Bonferroni correction.

Single-cell RNA-seq data have been deposited in the Gene Expression Omnibus under the accession number GSE210226.

### Monocyte adoptive transfer

Bone marrow monocytes from UBI-GFP mice were isolated using the mouse Monocyte Isolation Kit (BM) from Miltenyi (130-100-629). 10^6^ monocytes in HBSS were injected iv. into C57BL/6J mice and recipient mice were sacrificed for blood collection at 4h and 24h post-transfer.

### Chemotaxis assay

Monocyte chemotaxis was measured using the Incucyte ClearView 96 well chemotaxis plate in the Incucyte Live-cell Imaging System (Sartorius). 5000 JAM-A^+^ or JAM-A^-^ monocytes sorted from the blood of Py8119 tumor-bearing mice were plated in the top well coated with 50 μg/ml Growth Factor Reduced Matrigel (Corning). The bottom well contained 100 ng/ml mouse recombinant CCL2 (R&D Systems). The medium used for both the top and bottom wells was RPMI with 10% (v/v) FCS, 300 μg/ml L-glutamine, 100 units/ml penicillin, 100 μg/ml streptomycin, 1% (v/v) MEM non-essential amino acids (Gibco), 1 mM sodium pyruvate (Gibco), 0.02 mM 2-mercaptoethanol (Sigma). Monocyte chemotaxis was measured by detecting the decrease in total cell area in the top well every 2 h as cells migrated towards CCL2 in the bottom well.

### Monocyte adhesion assay

Monocytes were sorted from the blood of Py8119 tumor-bearing mice and labeled with Calcein AM (Biolegend) following the manufacturer’s instructions. Labeled monocytes were plated in a 96 well plate on a monolayer of E2 endothelial cell line (20) and incubated for 1 h at 37°C. For activation of endothelial cells, 100 ng/ml mouse recombinant IL1β (R&D Systems) was added 5 hours prior to the addition of monocytes. After 1 h adhesion, the fluorescence signal at the bottom of the plate was measured at 520 nm (total fluorescence). The plate was then gently washed five times with prewarmed phenol red-free RPMI (Gibco) using a multichannel pipette. Fluorescence signal at the bottom of the plate was measured again at 520 nm (adherent fluorescence). The percentage of adherent monocytes was calculated as the percentage of adherent fluorescence signal compared to the total fluorescence signal.

### IL1β ELISA

IL1β production in cell culture supernatants was measured using ELISA following the manufacturer’s instructions (R&D Systems, MLB00C). ELISA measurements were obtained using a VersaMax microplate reader (Molecular Devices) set to 450 nm. Measurements at 540 nm were used for background correction.

### Detection of monocyte-platelet aggregates

Detection of monocyte-platelet aggregates was performed based on published protocols (21, 22). 3 µl blood was collected from the tail vein and diluted in 90 µl HBSS with 0.5% FBS. After blocking Fcγ receptors (see above), fluorescently labeled antibodies were added to stain for leukocyte markers (CD45, CD11b, Ly6C, Ly6G, MHC-II, see antibody clones above) together with platelet markers CD41 (clone MWReg30) and CD42d (clone 1C2). After 20 minutes of incubation in the dark at 4°C, the samples were diluted to 400 µl. 7-AAD was added to exclude dead cells, and the samples were acquired using BD FACSCanto II (BD Biosciences) flow cytometer using low flow rate.

### Statistical analysis

Statistical analyses were performed in GraphPad Prism software and R as described in the corresponding sections of Materials and methods and figure legends. For relevant pairwise comparisons, paired or unpaired two-tailed t-test was used to calculate the *P* value as indicated in the figure legends. A *P* value < 0.05 was considered statistically significant. For statistically significant differences, the *P* value is indicated in graphs as the following: *P< 0.05, **P< 0.01, ***P< 0.001, ****P < 0.0001. P values for comparisons found to be nonsignificant are not shown.

## Results

### Cancer-associated inflammation promotes upregulation of JAM-A on peripheral monocytes in an IL-1β-dependent manner

**Figure 1:**
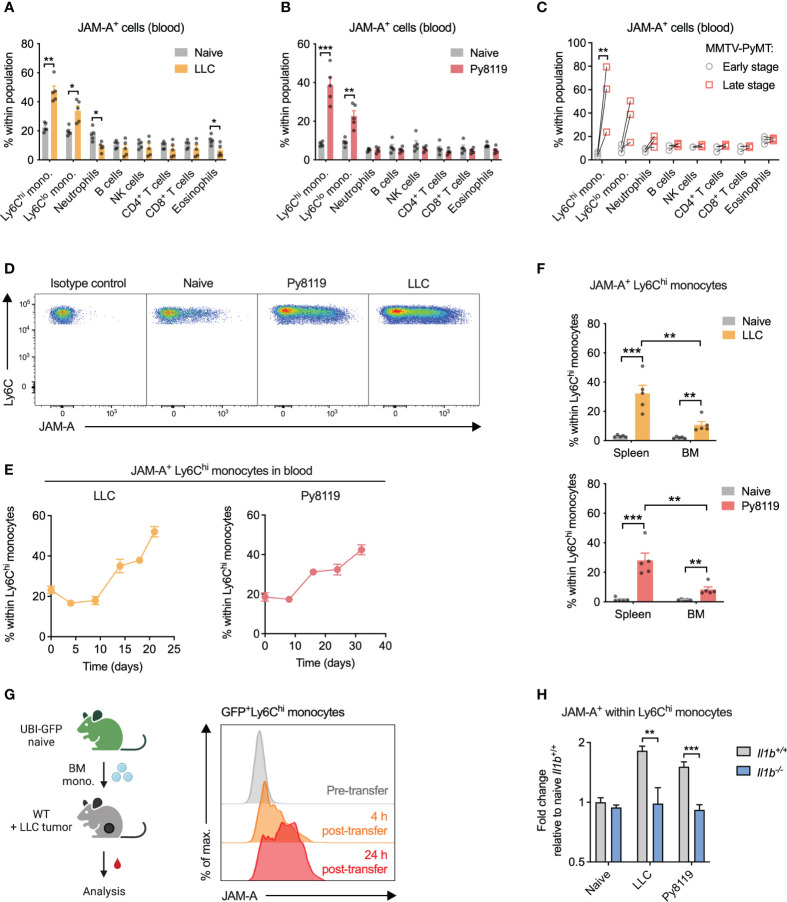
Cancer-associated inflammation promotes upregulation of JAM-A on peripheral monocytes in an IL-1β-dependent manner. **(A)** Frequency of JAM-A^+^ cells within the most abundant immune cell populations in the blood of mice with sc. LLC tumors (21 days post-engraftment, 1678 ± 212mm^3^ tumor volume). **(B)** Same as **(A)** in orthothopic Py8119 breast tumors (32 days post-engraftment, 1199 ± 114mm^3^ tumor volume). **(C)** Frequency of JAM-A^+^ cells within the most abundant immune cell populations in the blood of MMTV-PyMT mice at early stage (no palpable tumor) and late stage (tumor volume reached 1500 mm^3^) of tumor progression. Dots connected with line denote values from the same animal. **(D)** Representative flow cytometry dot plots of JAM-A expression in Ly6C^hi^ monocytes from the blood. **(E)** Changes in the frequency of JAM-A^+^ cells within Ly6C^hi^ monocytes during tumor progression in the blood of mice with orthotopic Py8119 tumors or sc. LLC tumors (corresponding tumor volumes are shown in **Supplementary Figure 2**). **(F)** Frequency of JAM-A^+^ cells within Ly6C^hi^ monocytes in the spleen and bone marrow (BM) of mice with orthotopic Py8119 tumors or sc. LLC tumors (time post-engraftment and tumor volume as in panels **A** and **B**). **(G)** JAM-A expression on GFP^+^ Ly6C^hi^ bone marrow-derived monocytes adoptively transferred into LLC tumor-bearing mice on day 18 of tumor development. **(H)** Fold change of JAM-A^+^ cells within Ly6C^hi^ monocytes in the blood of *Il1b* ^+/+^ or *Il1b* ^-/-^ mice with sc. LLC tumors (day 19 post-engraftment) or orthotopic Py8119 tumors (day 35 post-engraftment) compared to naive *Il1b* ^+/+^ mice (tumor burden is shown in **Supplementary Figure 3**). Cell type frequencies were determined using flow cytometry. Graphs show mean and SEM. Panel **(C)** paired two-tailed t-test; All other panels: unpaired two-tailed t-test; *P < 0.05, **P < 0.01, ***P < 0.001.

Considering that JAM-A reportedly promotes extravasation of circulating leukocytes to tissues (23, 24), we first wanted to determine which immune cell types express JAM-A in the circulation and whether JAM-A expression on these cells is altered in cancer. To this end, we examined surface JAM-A expression by flow cytometry across the most abundant immune cell types in the blood of healthy and tumor-bearing mice. In the steady-state, 10-20% of cells showed JAM-A expression in all examined immune cell types in the blood (**Figures 1A–C**, see **Supplementary Figure 1** for gating strategy). When examining the blood of mice bearing syngeneic subcutaneous Lewis lung carcinoma (LLC) tumors, we found that the proportion of JAM-A^+^ cells increased selectively among Ly6C^hi^ and Ly6C^lo^ monocytes, but not in other immune cell types (**Figures 1A, D**). Similar selective expansion of JAM-A^+^ monocytes in the circulation was observed upon implanting syngeneic Py8119 breast cancer cells (derived from MMTV-PyMT tumors) into the mammary fat pad (**Figures 1B, D**). To confirm that these findings were not specific for implantable tumor models, we repeated the same analysis in transgenic MMTV-PyMT mice that spontaneously develop breast adenocarcinomas. Indeed, the frequency of JAM-A^+^ monocytes increased significantly between early-stage disease and late-stage disease (**Figure 1C**). Ly6C^hi^ monocytes have been shown to infiltrate tumors and give rise to macrophages, whereas Ly6C^lo^ monocytes remain dominantly intravascular (25–28). Therefore, we focused our subsequent analyses on the Ly6C^hi^ subset as a key circulating precursor of tumor-promoting macrophages.

The proportion of JAM-A^+^ cells within circulating Ly6C^hi^ monocytes showed a gradual increase during tumor progression in both Py8119 and LLC tumor-bearing mice, suggesting that upregulation of JAM-A is dictated by the tumor burden (**Figure 1E**; **Supplementary Figure 2**). Expansion of JAM-A^+^ monocytes was also observed in the spleen and, to a lesser extent, in the bone marrow of tumor-bearing mice (**Figure 1F**). To confirm that JAM-A^+^ cells are *bona fide* monocytes, we isolated GFP^+^JAM-A^-^ monocytes from the bone marrow of healthy UBI-GFP mice and transferred them intravenously into LLC tumor-bearing mice. Analysis of the blood of recipient mice revealed that transferred monocytes acquired JAM-A expression as early as 4 hours after transfer in the circulation and this subset continued to expand 24 hours post-transfer (**Figure 1G**).

Systemic low-grade inflammation has been shown to be associated with enhanced mRNA level expression of JAM-A in circulating leukocytes (29). We and others have previously shown in several different mouse models that the presence of a tumor leads to elevated serum levels of the pro-inflammatory cytokine IL1β (30, 31). Hence, we hypothesized that IL1β may be important in driving the expansion of JAM-A^+^ monocytes. To test this hypothesis, we implanted LLC or Py8119 tumors in IL1β-deficient mice and assessed the frequency of JAM-A^+^ cells within Ly6C^hi^ monocytes. As shown in **Figure 1H**, deletion of IL1β prevented tumor-induced expansion of JAM-A^+^ cells within monocytes in both LLC and Py8119 tumor-bearing mice. Reduced expansion of JAM-A^+^ cells was likely not due to different tumor burden in *Il1b* ^-/-^ mice, as only LLC tumors but not Py8119 tumors showed altered tumor weight in IL1β-deficient mice (**Supplementary Figure 3**). Lower JAM-A expression in *Il1b* ^-/-^ mice was associated with reduced circulating monocyte frequency in the Py8119 model, but not in LLC, suggesting that JAM-A expression and monocyte mobilization were independently regulated (**Supplementary Figure 4**).

We hypothesized that JAM-A^+^Ly6C^hi^ monocytes may represent a functionally distinct population in terms of migratory or adhesive capacity, two key JAM-A-regulated processes. JAM-A^+^ monocytes from tumor-bearing mice showed an increase in surface but not in total β_1_ integrin levels, whereas β_2_ integrin could not be detected (**Supplementary Figure 5A**). This indicated preferential membrane localization of β_1_ integrin in JAM-A^+^ monocytes.

Nevertheless, *in vitro* assays did not reveal major differences between JAM-A^-^ and JAM-A^+^ monocytes neither in their migration capacity towards CCL2 nor in their adhesion capacity to an endothelial monolayer (**Supplementary Figures 5B, C**).

Altogether, these data show that IL1β-driven systemic low-grade inflammation in cancer enhances JAM-A expression on circulating monocytes.

### JAM-A is expressed on mononuclear phagocytes infiltrating human and mouse tumors

**Figure 2:**
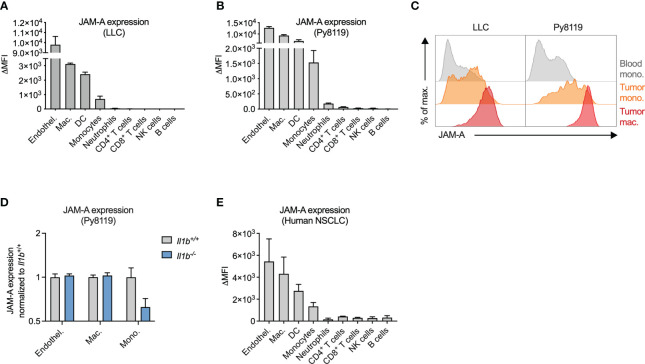
JAM-A is expressed on mononuclear phagocytes infiltrating human and mouse tumors. **(A)** JAM-A expression on endothelial cells and immune cells in LLC tumors (n=4-5). 21 days post-engraftment, 1678 ± 212mm^3^ tumor volume. **(B)** Same as **(A)** in Py8119 tumors (n=4-6). 32 days post-engraftment, 1199 ± 114mm^3^ tumor volume. **(C)** Representative histogram of JAM-A expression on blood monocytes, tumor-infiltrating monocytes and tumor-associated macrophages in LLC and Py8119 tumors. **(D)** Fold change of JAM-A expression on macrophages, monocytes and endothelial cells in Py8119 tumors of *Il1b* ^-/-^ mice compared to *Il1b* ^+/+^ mice (n=6). 35 days post-engraftment, tumor burden is shown in **Supplementary Figure 3**. **(E)** JAM-A expression on endothelial cells and immune cells in human non-small cell lung cancer (NSCLC) (n=3). JAM-A expression was determined using flow cytometry. ΔMFI: Delta Median Fluorescent Intensity (MFI) = MFI of anti-JAM-A-stained sample minus MFI of isotype control-stained sample. Bar graphs show mean and SEM.

Next, we wanted to determine whether JAM-A expression on monocytes persists upon tumor infiltration and differentiation into macrophages and whether additional myeloid cell types in the tumor express this protein. To this end, we examined cell surface JAM-A expression *via* flow cytometry on immune cells infiltrating LLC and Py8119 tumors (**Figures 2A, B**). We used endothelial cells as a reference cell type, which expressed high levels of JAM-A, in agreement with the literature (**Figures 2A, B**) (32). This analysis revealed high levels of JAM-A expression on macrophages and dendritic cells (DCs), and intermediate expression levels on monocytes in both LLC and Py8119 tumors (**Figures 2A, B**). Neutrophils and lymphoid cells, on the other hand, showed lower or no JAM-A expression (**Figures 2A, B**). When compared to circulating monocytes, tumor-infiltrating monocytes and macrophages showed a gradual increase in surface JAM-A expression suggesting that JAM-A is upregulated during monocyte-to-macrophage differentiation, consistent with previous *in vitro* observations (**Figure 2C**) (33). In contrast to monocytes, macrophages and endothelial cells from tumors of *Il1b* ^-/-^ mice showed comparable JAM-A expression to wild-type controls, indicating that expression of JAM-A in these cells was not modulated by IL1β within the tumor microenvironment (**Figure 2D**). Abundance of both monocytes and macrophages in Py8119 tumors of *Il1b* ^-/-^ mice remained unaffected (**Supplementary Figure 6**). Notably, neither LLC nor Py8119 cancer cells secreted IL1β (reported in Kiss et al. (30) and shown in **Supplementary Figure 7**, respectively), hence cancer cells could not compensate for the knock-out of *Il1b* in tumor-infiltrating immune cells.

To assess JAM-A expression in myeloid cells infiltrating human tumors, we collected surgical tumor specimens from non-small cell lung cancer (NSCLC) patients and examined cell surface JAM-A expression *via* flow cytometry (**Figure 2E**, see **Supplementary Figure 1** for gating strategy). Similar to mouse tumors, JAM-A expression among human tumor-infiltrating immune cells was highest on myeloid cells: macrophages, DCs and monocytes (the latter cell population likely including immune-suppressive monocytes, also termed monocytic myeloid-derived suppressor cells). Thus, mouse tumors closely recapitulated human disease with regard to the expression pattern of JAM-A among immune cells, confirming the relevance of the models used.

Overall, these results show that JAM-A is primarily expressed on mononuclear phagocytes, including monocytes, macrophages and DCs, both in human and mouse tumors.

### JAM-A is dispensable for myeloid cell accumulation in the primary tumor and in the pulmonary metastatic site

**Figure 3:**
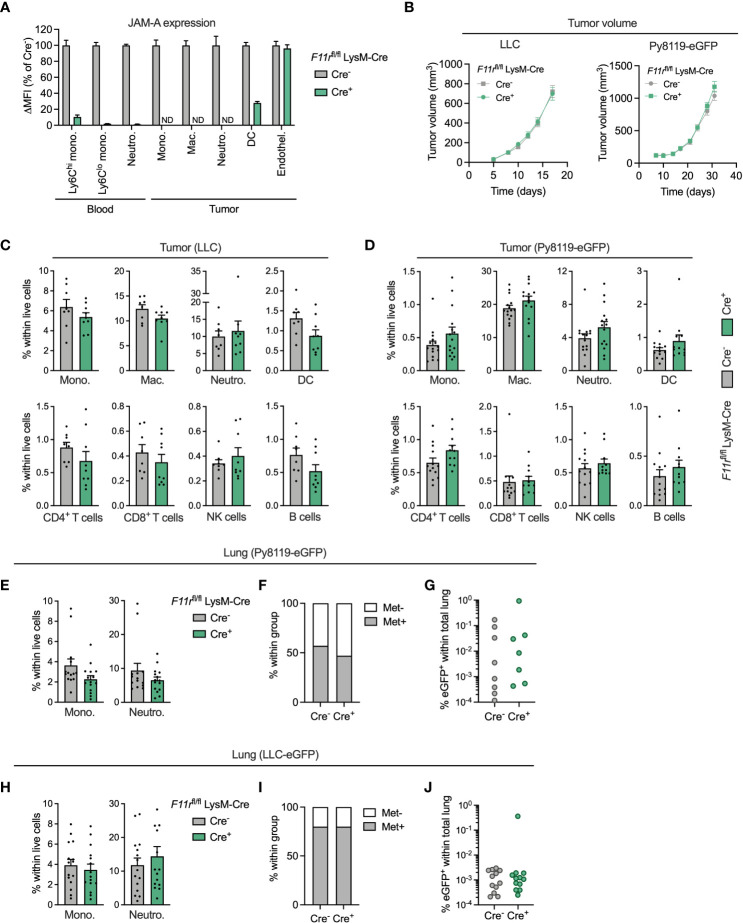
JAM-A is dispensable for myeloid cell accumulation in the primary tumor and in the pulmonary metastatic site. **(A)** JAM-A expression in myeloid cell populations and endothelial cells in LLC tumors of *F11r* ^flox/flox^ LysM-Cre^+^ mice normalized to *F11r* ^flox/flox^ LysM-Cre^-^ mice. ND = not detected. Day 17 post-engraftment, tumor volume shown in panel **(B)**. **(B)** LLC (n=16-17) and Py8119-eGFP (n=14-15) tumor growth in *F11r* ^flox/flox^ LysM-Cre^-^ and *F11r* ^flox/flox^ LysM-Cre^+^ mice. **(C)** Frequency of indicated myeloid and lymphoid cell populations in LLC tumors (n=9-10). Day 17 post-engraftment, tumor volume is shown in panel **(B)**. **(D)** Same as **(C)** in Py8119-eGFP tumors (n=12-13). Day 31 post-engraftment, tumor volume is shown in panel **(B)**. **(E)** Frequency of monocytes and neutrophils in the lungs of tumor-bearing mice with Py8119-eGFP tumors (n=14-15). Panel E-G: Day 31 post-engraftment. **(F)** Proportion of mice with eGFP^+^ metastatic Py8119 cancer cells in the lung detected *via* flow cytometry. **(G)** Frequency of eGFP^+^ metastatic cancer cells in the lungs of Py8119-eGFP tumor-bearing mice (n=14-15). **(H–J)** Same as **(E–G)** for LLC-eGFP tumors (n=15). Day 17 post-engraftment. JAM-A expression and cell frequencies were determined using flow cytometry. All graphs show mean and SEM.

To interrogate the function of JAM-A specifically in myeloid cells, we generated myeloid-specific JAM-A knock-out mice by crossing mice carrying floxed alleles of the JAM-A-encoding gene *F11r* with mice carrying the myeloid-specific LysM-Cre transgene (*F11r* ^flox/flox^;LysM-Cre^+^, hereafter *F11r* ^fl/fl^Cre^+^). These mice showed at least 90% reduction in JAM-A expression in circulating monocytes and neutrophils compared to control *F11r* ^flox/flox^;LysM-Cre^-^ mice (hereafter *F11r* ^fl/fl^Cre^-^) (**Figure 3A**). In the tumor, JAM-A was reduced to undetectable levels in monocytes, macrophages and neutrophils and was 72% lower in dendritic cells (**Figure 3A**). JAM-A expression remained unaffected in tumor endothelial cells of *F11r* ^fl/fl^Cre^+^ mice, allowing us to specifically address the role of myeloid cell-expressed JAM-A in the tumor microenvironment (**Figure 3A**). We chose to examine the impact of myeloid-specific JAM-A deletion in the syngeneic LLC and Py8119 tumor models because these tumors are heavily infiltrated by myeloid cells, similar to the majority of human lung and breast tumors (34, 35). Both tumor models have been shown to rely on the recruitment of monocytes and neutrophils for their progression (30, 36, 37). Hence, we hypothesized that JAM-A in myeloid cells may be required for this process.

Myeloid-specific deletion of JAM-A in *F11r* ^fl/fl^Cre^+^ mice did not affect subcutaneous LLC or orthotopic Py8119-eGFP tumor growth (**Figure 3B**). Surprisingly, analysis of the tumor immune microenvironment did not reveal significant differences in the infiltration of monocytes, macrophages, neutrophils and DCs upon myeloid JAM-A deletion in either tumor type (**Figures 3C, D**). The proportion of T cells, NK cells and B cells also remained unaffected in *F11r* ^fl/fl^Cre^+^ mice (**Figures 3C, D**).

Monocytes and neutrophils accumulate in metastatic sites, most notably in the lung, and can promote outgrowth of disseminated cancer cells (38, 39). Thus, we also examined whether JAM-A deletion affects myeloid cell recruitment to the pulmonary metastatic site. We found that the relative frequency of monocytes and neutrophils was not affected by myeloid JAM-A deletion in the lung of mice with Py8119-eGFP breast tumors (**Figure 3E**). Accordingly, the proportion of mice with detectable GFP^+^ cancer cells and the abundance of metastatic cancer cells in the lung were comparable between *F11r* ^fl/fl^Cre^+^ mice and *F11r* ^fl/fl^Cre^-^ mice (**Figures 3E–G**). Similar results were obtained in mice with subcutaneous LLC-eGFP tumors (**Figures 3H–J**). Monocyte accumulation in tumor-draining lymph nodes of LLC-eGFP tumors was similarly unaltered (**Supplementary Figure 8**).

JAM-A is also expressed by platelets (40). Altered monocyte-platelet interactions upon myeloid-specific JAM-A deletion could potentially confound our *in vivo* observations. We excluded this possibility by confirming that JAM-A deletion did not influence the frequency of monocyte-platelet aggregates in the blood (**Supplementary Figure 9**).

Collectively, these results indicate that JAM-A expressed by myeloid cells is not critically required for their accumulation in the primary tumor and in the pulmonary metastatic site.

### JAM-A does not affect myeloid cell diversification in the tumor microenvironment

**Figure 4:**
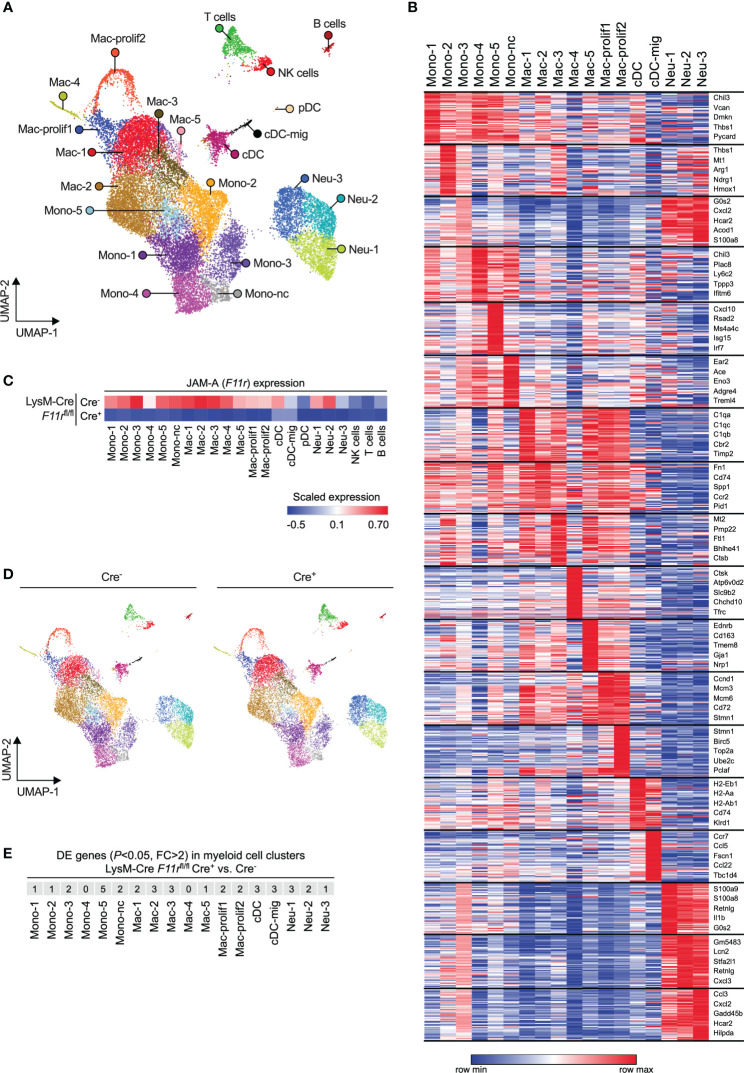
JAM-A does not affect myeloid cell diversification in the tumor microenvironment. **(A)** UMAP plot showing CD45^+^ immune cells from LLC tumors of both *F11r* ^flox/flox^ LysM-Cre^-^ and *F11r* ^flox/flox^ LysM-Cre^+^ mice color-coded by clusters defined based on single-cell RNA-seq data. **(B)** Heatmap showing the top 50 enriched genes in each myeloid cell cluster. The top 5 enriched genes per cluster are highlighted. See **Supplementary Table 1** for list of top 50 enriched genes for all clusters shown in panel **(A)**. **(C)** Heatmap showing *F11r* (encoding JAM-A) expression in tumors of *F11r* ^flox/flox^ LysM-Cre^-^ and *F11r* ^flox/flox^ LysM-Cre^+^ mice based on single-cell RNA-seq data. **(D)** UMAP plots showing CD45^+^ immune cells from LLC tumors of *F11r* ^flox/flox^ LysM-Cre^-^ or *F11r* ^flox/flox^ LysM-Cre^+^ mice color-coded by clusters shown in panel **(A)**. **(E)** Number of differentially expressed (DE) genes (adjusted *P* value < 0.05, fold change (FC) > 2) between *F11r* ^flox/flox^ LysM-Cre^+^ and *F11r* ^flox/flox^ LysM-Cre^-^ mice within each myeloid cell cluster. See **Supplementary Table 2** for full list of DE genes.

Receptors that mediate interactions with endothelial cells are not only important for transmigration but can also shape the transcriptome and thereby the functionality of myeloid cells upon tissue infiltration (41, 42). Hence, JAM-A could presumably affect the phenotype of myeloid cells either directly through activating downstream signaling pathways *via* its intracellular domain or indirectly by modulating integrin signaling (11, 43). Hence, we examined whether JAM-A deletion has an impact on the transcriptome of tumor-infiltrating myeloid cells by performing single-cell RNA sequencing of CD45^+^ immune cells sorted from size-matched LLC tumors of *F11r* ^fl/fl^Cre^-^ and *F11r* ^fl/fl^Cre^+^ mice. Unsupervised analysis of single cell transcriptomes in the combined *F11r* ^fl/fl^Cre^-^ and *F11r* ^fl/fl^Cre^+^ dataset revealed 22 cell clusters which we annotated based on cell type-specific gene expression (**Figures 4A, B**; **Supplementary Figure 10**; **Supplementary Table 1**). Among myeloid cells, which comprised the majority of cells, we found 6 monocyte states (including nonclassical monocytes labeled as Mono-nc) and 7 macrophage states (including 2 proliferating macrophage states labeled as Mac-prolif1/2) (**Figures 4A, B**). We also identified plasmacytoid DCs (pDC) and conventional DCs (cDC) as well as cDCs with high *Ccr7* expression corresponding to activated migratory cDCs (cDC-mig) (**Figures 4A, B**). Neutrophils partitioned into 3 clusters (Neu-1,2,3) which represented a continuum of transcriptional states rather than highly distinct populations (**Figures 4A, B**).

Examining JAM-A (*F11r*) expression across the various cell states confirmed our observations that JAM-A is primarily expressed in myeloid cells and that LysM*-*Cre causes strong reduction of *F11r* levels in these cells (**Figure 4C**). Two of the three tumor-infiltrating neutrophil states showed relatively high expression of *F11r* mRNA despite low overall JAM-A protein expression in neutrophils (**Figures 4C, 2A, B**). Interestingly, the migratory subset of cDCs showed lower *F11r* expression compared to the rest of cDCs, which is in line with the prior observation that the absence of JAM-A allows for more efficient DC migration to the lymph node (**Figure 4C**) (42). One neutrophil state (Neu-1) and all monocyte states, with the exception of Mono-2, expressed the subunits of LFA-1 (α_L_β_2_) integrin in both *F11r* ^fl/fl^Cre^-^ and *F11r* ^fl/fl^Cre^+^ mice, suggesting that these cells could potentially interact with endothelial JAM-A *via* LFA-1 (**Supplementary Figure 11**).

Next, we asked the question whether JAM-A deletion could prevent the acquisition of any of the myeloid cell states we identified. Comparison of the population structure between *F11r* ^fl/fl^Cre^-^ and *F11r* ^fl/fl^Cre^+^ mice showed that all monocyte, macrophage, neutrophil and DC transcriptional states observed in the tumors of control mice could also be found in myeloid JAM-A-deficient mice (**Figure 4D**). Hence, JAM-A was not required for the induction of specific myeloid cell transcriptional states upon tumor infiltration.

Finally, we determined whether JAM-A deletion caused changes in gene expression within the various myeloid cell states. Using the rather permissive fold change threshold of log(2)0.5 (≈1.4), we found less than 10 differentially expressed genes in most myeloid states (**Supplementary Table 2**). Furthermore, we found that in all myeloid cell states only 5 or less genes showed fold changes higher than log(2)1 (=2.0) upon JAM-A deletion (**Figure 4E**).

It has been previously reported that the genes *Retnla* and *Ifi202b* are upregulated in JAM-A-deficient brain macrophages (12). We found that neither *Retnla* nor *Ifi202b* expression was altered in any of the tumor-infiltrating myeloid states by JAM-A deletion (**Supplementary Figure 11**).

Together, these results demonstrate that JAM-A deletion does not prevent the acquisition of diverse myeloid cell states upon tumor infiltration and does not cause major gene expression changes in these cells.

## Discussion

Evidence from both animal models and patients with cancer have demonstrated that solid tumors can have an impact on the systemic immune environment, which can contribute to disease progression and may increase the risk of certain comorbidities (44). This involves cancer-induced alterations in the phenotype of circulating monocytes, including changes in surface protein expression, cytokine secretion and transcriptome (45).

The data presented here demonstrating that JAM-A expression in circulating monocytes increases in response to a distant tumor further support this notion. As tumor-induced JAM-A upregulation was IL1β-dependent, our findings are likely relevant for other diseases associated with elevated systemic IL1β levels. Accordingly, a previous study showed upregulation of the *F11R* gene encoding JAM-A in peripheral blood mononuclear cells from patients with rheumatoid arthritis, a condition characterized by high IL1β release (29). Despite the reported roles for JAM-A in cell migration and adhesion, JAM-A^+^ monocytes isolated from the blood of tumor-bearing mice did not show more efficient migration or adhesion *in vitro*. However, intravital imaging could potentially reveal whether their dynamic *in vivo* behavior could be altered. It also remains to be determined whether tumor-induced JAM-A expression could facilitate monocyte recruitment to sites of inflammation where JAM-A was shown to be essential, such as atherosclerotic plaques or the ischemic myocardium (2, 3).

We unexpectedly found that JAM-A was not critically required for myeloid cell accumulation in tumors. This is in contrast with observations made in mouse models of peritonitis, ischemia-reperfusion injury and atherosclerosis which showed reduced monocyte and neutrophil recruitment to sites of inflammation when wild-type mice were transplanted with JAM-A-deficient bone marrow, resulting in leukocyte-specific JAM-A deletion (2, 3). This suggests that myeloid cells infiltrating tumors may utilize pathways that are distinct from those required for extravasation in inflammatory sites. It is also conceivable that overproduction of myeloid cell recruitment signals by the tumor bypasses the need for JAM-A, as opposed to inflammatory sites where myeloid cell recruitment is more strictly regulated. Further research will be needed to dissect the differences in myeloid cell recruitment mechanisms between inflammation and cancer as well as the mechanisms underlying this dichotomy.

A limitation of our study is that we assessed myeloid cell abundance in tumors of myeloid-specific JAM-A knock-out mice at late-stage disease, when JAM-A expression was highest on peripheral monocytes. JAM-A could potentially play a role in myeloid cell recruitment at early stages of the disease, followed by JAM-A-independent myeloid cell accumulation during tumor progression.

We found that JAM-A was highly expressed on tumor endothelial cells but we did not investigate whether its junctional localization was preserved in the tumor vasculature. However, the expression level and localization of endothelial JAM-A likely did not influence our results. According to previous studies using inflammation models, JAM-A-deficient monocytes and neutrophils showed stronger adhesion and deficient transmigration across both JAM-A-expressing and JAM-A-deficient endothelium (2, 3). It nevertheless remains to be elucidated whether endothelial JAM-A is required for myeloid cell accumulation in tumors by interacting with ligands on myeloid cells other than JAM-A, such as LFA-1 (7).

We showed in both human and mouse tumors that macrophages exhibited the highest JAM-A expression among the examined tumor-infiltrating immune cell types. It has been previously demonstrated that macrophages isolated from the brain of whole-body JAM-A-deficient mice show altered gene expression, enhanced phagocytosis and promote the proliferation of cancer cells *in vitro* (12). This was associated with accelerated glioma progression in JAM-A knock-out mice, specifically in females (12). Upon myeloid-specific JAM-A deletion in female mice, we did not observe major transcriptional changes in tumor-associated macrophages and disease progression remained unaltered in the LLC and Py8119 tumor models. Hence, the role of JAM-A in macrophages may depend on the tissue context. Alternatively, some changes in macrophage phenotype previously observed in JAM-A-deficient mice may be secondary effects of whole-body JAM-A deletion. Such secondary effects on myeloid cell activity were demonstrated by a recent study: septic whole-body JAM-A knock-out mice generate an augmented B cell response and higher systemic levels of opsonizing IgA antibodies, which, in turn, leads to enhanced phagocytosis of bacteria by circulating neutrophils (16).

In summary, we show in two distinct mouse models that JAM-A expression in myeloid cells is dispensable for their accumulation in tumors, despite previous studies demonstrating a role for this protein supporting myeloid cell extravasation in other disease contexts. We found that tumors nevertheless upregulate JAM-A expression on circulating monocytes, which could potentially influence monocyte recruitment to other tissue sites. In contrast to previous findings in whole-body JAM-A knock-out mice, we did not observe transcriptional alterations in macrophages when we deleted JAM-A specifically in myeloid cells. Altogether, these results will inform future studies on the cell-specific roles of JAM-A in cancer and inflammatory diseases as well as the development of JAM-A-targeted therapies.

## Data availability statement

The datasets presented in this study can be found in online repositories. The names of the repository/repositories and accession number(s) can be found below: Gene Expression Omnibus *via* accession ID: GSE210226.

## Ethics statement

The studies involving human participants were reviewed and approved by ethics committees of the Vrije Universiteit Brussel and Saint-Luc-UCLouvain. The patients/participants provided their written informed consent to participate in this study. The animal study was reviewed and approved by Ethical Committee for Animal Experiments of Vrije Universiteit Brussel.

## Author contributions

MK: Designed and performed research, analyzed data, and wrote the manuscript; AM, HVD, EL, SA, and PB: performed research; DK: analyzed single cell RNA-seq data; IS: prepared libraries for single-cell RNA-seq; AT: performed chemotaxis assays; JB: performed research and mouse genotyping; YE: assisted with cell sorting; FA: provided clinical samples; RA and CG: generated eGFP-expressing cancer cell lines; JVG and DL: designed experiments, supervised and coordinated research, provided funding, edited the manuscript. All authors contributed to the article and approved the submitted version.

## Funding

MK, AM, HVD, EL, SA, and PB are supported by doctoral grants from Research Foundation Flanders (FWO, grant numbers 1S23316N, 1S16718N, 1S24117N, 1S67419N, 1S78120N, 1154720N). MK and AM are supported by doctoral grants from Kom op Tegen Kanker (Stand up to Cancer), the Flemish Cancer Society. DL is supported by grants from FWO (12Z1820N), Kom op Tegen Kanker and Vrije Universiteit Brussel. JVG is supported by FWO, Kom op Tegen Kanker, Stichting tegen kanker and Vrije Universiteit Brussel.

## Acknowledgments

The authors thank Prof. Massimiliano Mazzone for providing the C57BL/6-MMTV-PyMT mice.

## Conflict of interest

The authors declare that the research was conducted in the absence of any commercial or financial relationships that could be construed as a potential conflict of interest.

## Publisher’s note

All claims expressed in this article are solely those of the authors and do not necessarily represent those of their affiliated organizations, or those of the publisher, the editors and the reviewers. Any product that may be evaluated in this article, or claim that may be made by its manufacturer, is not guaranteed or endorsed by the publisher.
