# Supplementary material for: Junctional adhesion molecule-A is dispensable for myeloid cell recruitment and diversification in the tumor microenvironment
Source: Front Immunol. 2022 Dec 1;13:1003975. doi: 10.3389/fimmu.2022.1003975 (PMC9751033; doi:10.3389/fimmu.2022.1003975)
Supplement: Supplementary file 3 [file DataSheet_1.pdf]

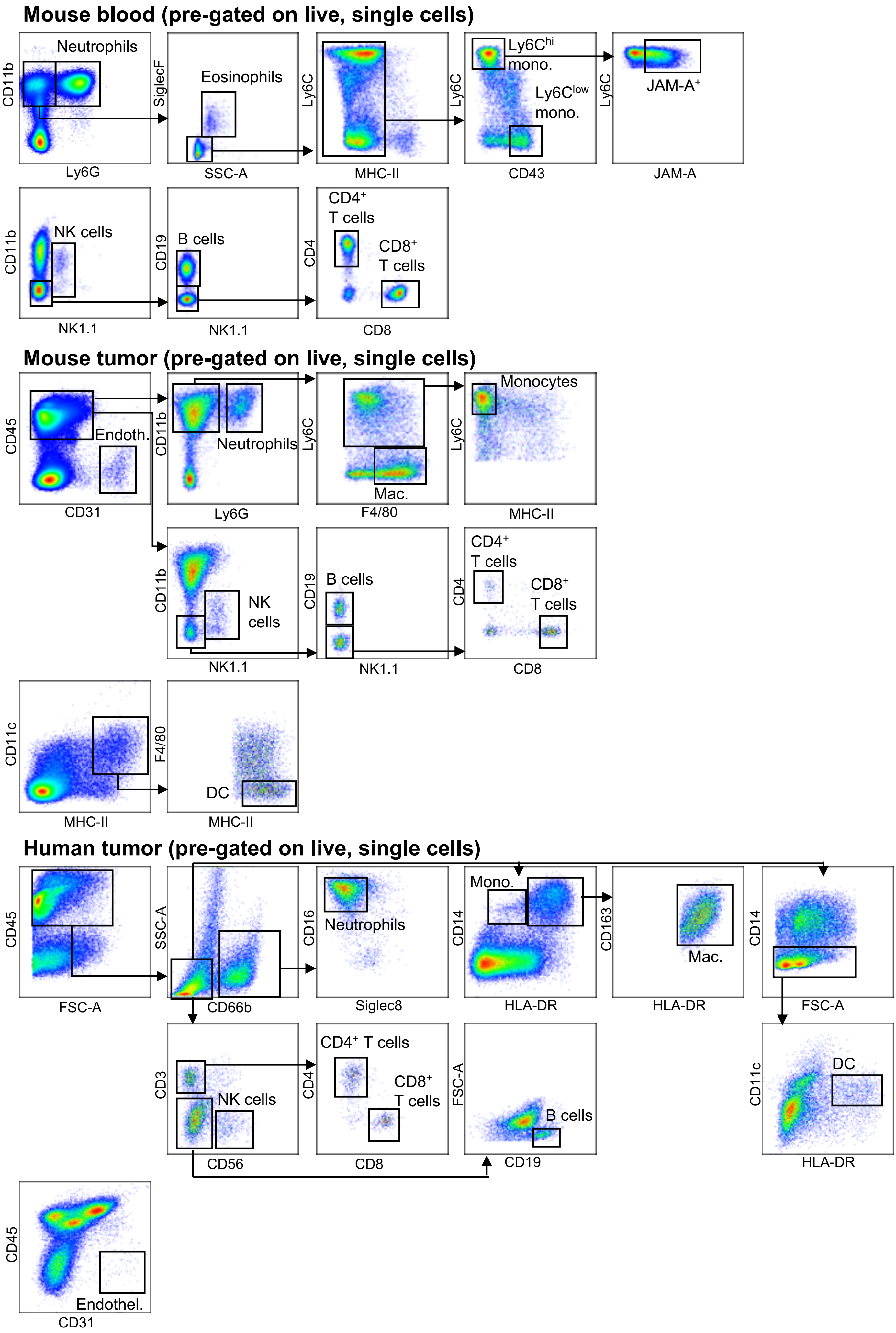

Supplementary Figure 1. Flow cytometry gating strategies used to identify cell populations

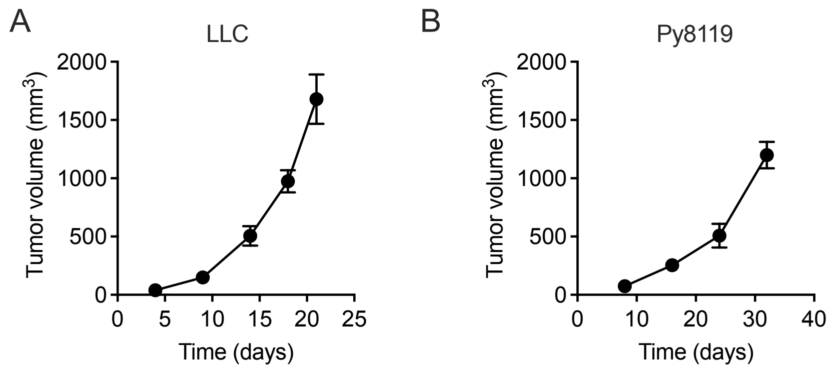

Supplementary Figure 2. Tumor volumes at the time points shown in Figure 1E
(A) Tumor volume of sc. LLC tumors (n=6) from experiment shown in Figure 1E
(B) Tumor volume of orthotopic Py8119 tumors (n=6) from experiment shown in Figure 1E
Graphs show mean and SEM.

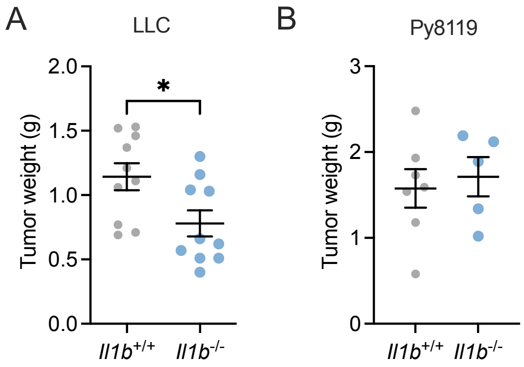

Supplementary Figure 3. Tumor burden in *Il1b*$^{-/-}$ mice

(A) LLC tumor weight in *Il1b*$^{+/+}$ and *Il1b*$^{-/-}$ mice (day 19 post-engraftment).

(B) Py8119 tumor weight in *Il1b*$^{+/+}$ and *Il1b*$^{-/-}$ mice (day 35 post-engraftment).

Graphs show mean and SEM.

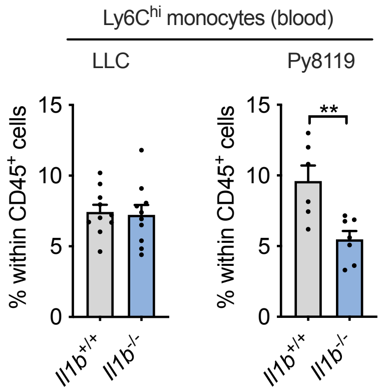

Supplementary Figure 4. Ly6C^{hi} monocyte frequency in the peripheral blood of *Il1b$^{-/-}$* mice
Frequency of Ly6C^{hi} monocytes in the peripheral blood of LLC (n=10) and Py8119 (n=6-7) tumor bearing *Il1b*$^{-/-}$ and *Il1b*$^{+/+}$ mice, assessed by flow cytometry. Graphs show mean and SEM.

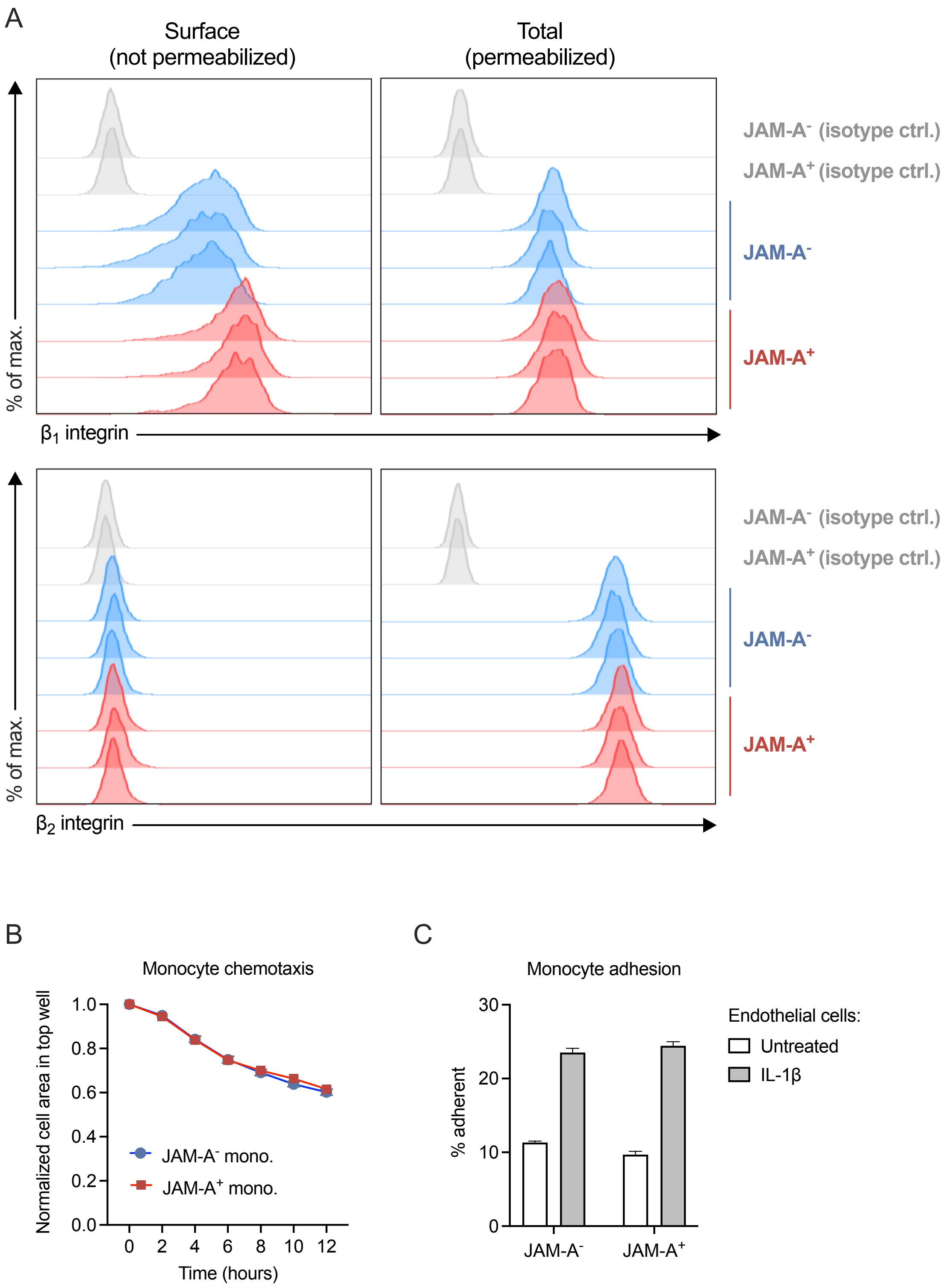

Supplementary Figure 5. Phenotype comparison of JAM-A+ and JAM-A- monocytes

(A) Expression of β_1 and β_2 integrins in peripheral blood JAM-A$^+$ and JAM-A$^-$ Ly6C^{hi} monocytes from Py8119 tumor-bearing mice stained with or without membrane permeabilization (for examining total or surface levels, respectively).

(B) Transwell migration of JAM-A$^+$ and JAM-A$^-$ Ly6C^{hi} monocytes isolated from the blood of Py8119 tumor-bearing mice (n=8). CCL2 was used as a chemotactic stimulus in the bottom well.

(C) Adhesion of JAM-A$^+$ and JAM-A$^-$ Ly6C^{hi} monocytes isolated from the blood Py8119 tumor-bearing mice (n=3). Proportion of adherent monocytes on untreated or IL1β-pretreated endothelial monolayers.

Integrin levels were assessed by flow cytometry. Graphs in panels B and C show mean and SEM.

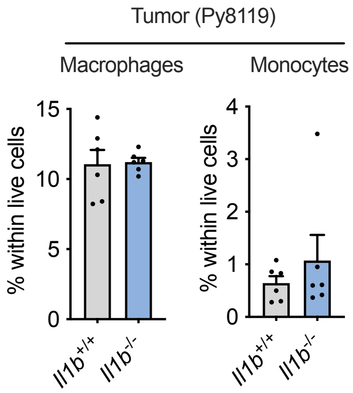

Supplementary Figure 6. Macrophage and monocyte abundance in tumors of *Il1b*$^{-/-}$ mice
Frequency of macrophages and monocytes in Py8119 tumors of *Il1b*$^{-/-}$ and *Il1b*$^{+/+}$ mice, assessed by flow cytometry (n=6). Graphs shows mean and SEM.

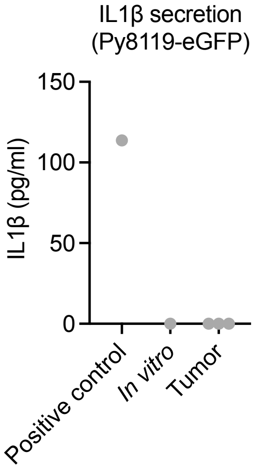

Supplementary Figure 7. Lack of IL1β secretion by Py8119 cancer cells
IL1β production by Py8119-eGFP cancer cells measured using ELISA. Positive control: Recombinant mouse IL1β; *In vitro*: Py8119-eGFP cell culture supernatant; Tumor: Supernatant of Py8119-eGFP cancer cells which were sorted from established orthotopic tumors and cultured for 24 hours.

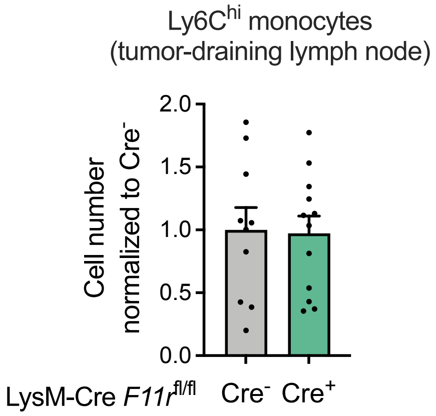

Supplementary Figure 8. Ly6C^{hi} monocyte abundance in the tumor-draining lymph node
Number of Ly6C^{hi} monocytes per tumor-draining lymph node determined by flow cytometry in LLC-eGFP tumor-bearing LysM-Cre *F11r*$^{fl/fl}$ mice, normalized to Cre^{-} controls (n=10/12, data pooled from two experiments). Graph shows mean and SEM.

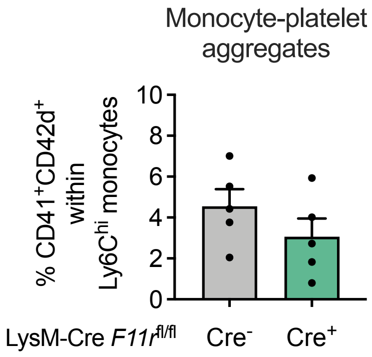

Supplementary Figure 9. Monocyte-platelet aggregates in myeloid-specific JAM-A-deficient mice

Frequency of monocytes in LLC-eGFP tumor-bearing LysM-Cre *F11r*$^{fl/fl}$ Cre^{-} and Cre^{+} mice associated with platelets assessed by flow cytometry staining of platelet markers (CD41 and CD42d) on the cell surface. Graph shows mean and SEM.

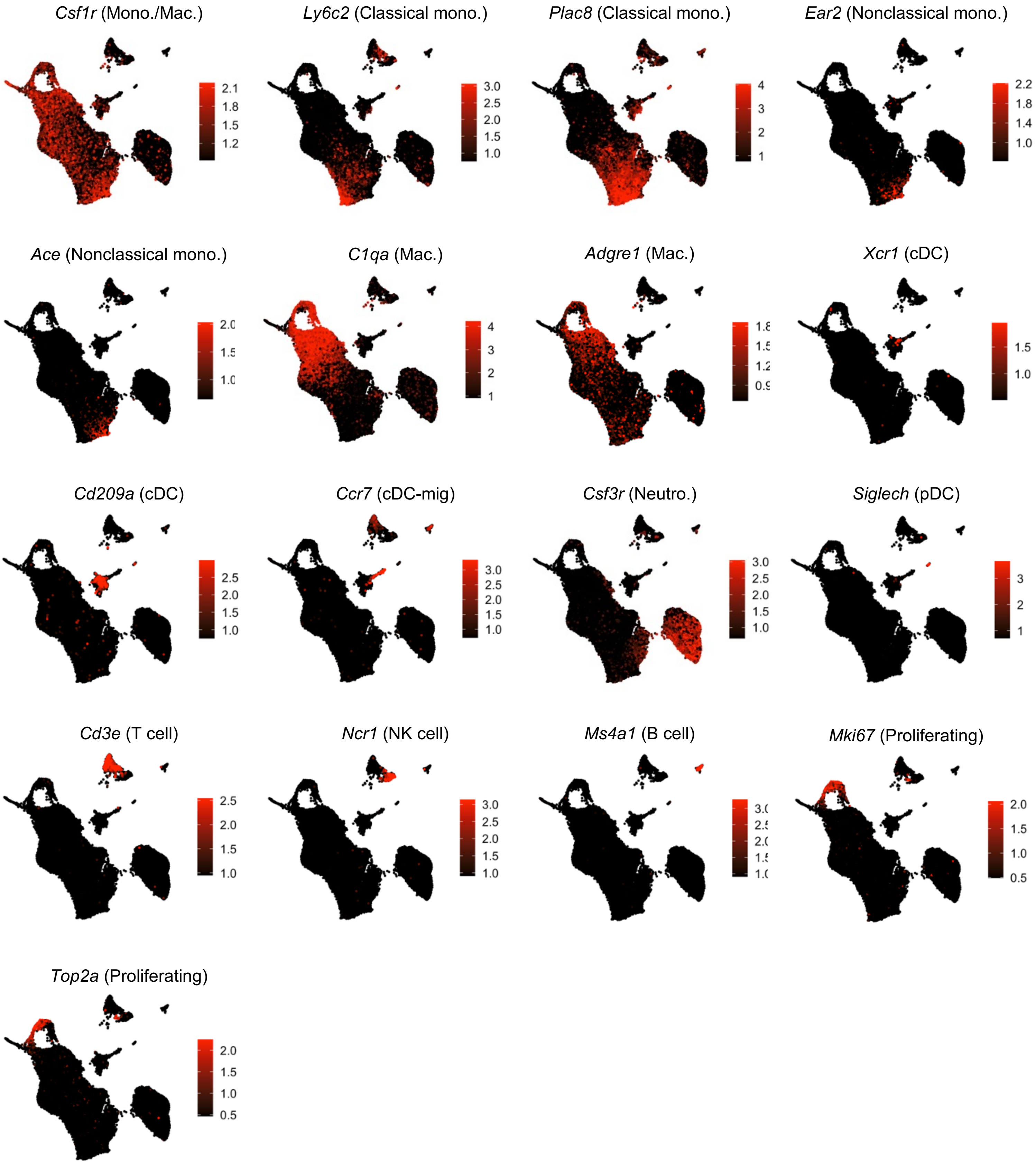

Supplementary Figure 10. UMAP plots colored based on expression of marker genes used to identify major cell populations in the single cell RNA-seq data

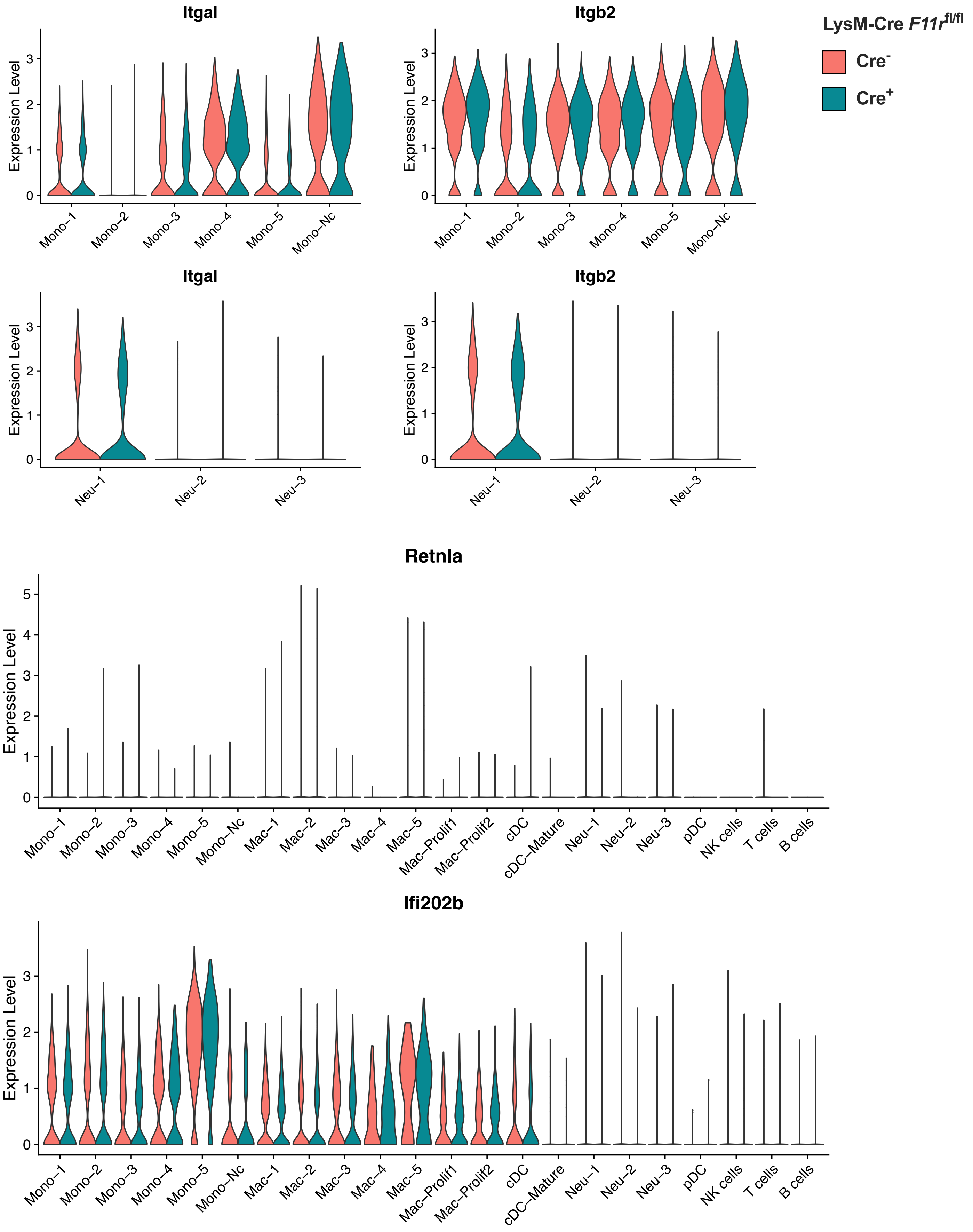

Supplementary Figure 11. Expression of *Itgal*, *Itgb2*, *Retnla* and *Ifi202b* in scRNAseq of LLC tumors

Expression of β2 integrin subunits (*Itgal*, *Itgb2***)** and previously reported JAM-A-regulated genes (*Retnla*, *Ifi202b*) across different immune cell states in LLC tumors of LysM-Cre *F11r*$^{fl/fl}$ Cre^{+} and Cre^{-} mice.
